# Recurrent huge leiomyoma of the urethra in a female patient: A case report

**DOI:** 10.3892/ol.2014.1991

**Published:** 2014-03-21

**Authors:** YUE-HONG SHEN, KAI YANG

**Affiliations:** Department of Urology, The First Affiliated Hospital, College of Medicine, Zhejiang University, Hangzhou, Zhejiang 310003, P.R. China

**Keywords:** leiomyoma, urethra

## Abstract

Urethral leiomyoma is an extremely rare condition that arises from the smooth muscle of the urethra. To the best of our knowledge, there is only a single reported recurrence treated by a repeat excision in the literature to date. The present study reports an exceptionally rare case of a recurrent huge leiomyoma of the female urethra. The 47-year-old female was diagnosed with a huge mass located between the urethra and vagina during a gynecological examination. The patient had no symptoms and was successfully treated with transabdominal excision. Pathological examinations revealed a leiomyoma of the urethra. The patient was followed up for one year without any sign of recurrence.

## Introduction

Urethral leiomyomas are rare mesenchymal benign tumors of smooth muscle origin that occur almost exclusively in females (1). To the best of our knowledge, only ~100 cases have been reported in the literature (1–8). The etiology and pathogenesis of these tumors are unclear, although, some are hypothesized to have a hormonal influence (5,6). Surgical excision is the first choice of treatment for these tumors and the prognosis has been good with the exception of one reported case of local recurrence (9). In the present study, a rare case of huge urethral leiomyoma recurrence in a Chinese female is reported. Patient provided written informed consent.

## Case report

**Figure 1:**
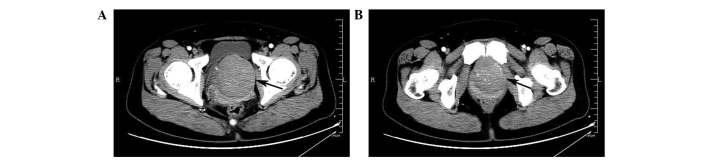
Contrast-enhanced computed tomography showing the presence of the tumor (A) compressing the bladder and uterine and (B) behind the pubic symphysis. Arrows indicate the tumor.

**Figure 2:**
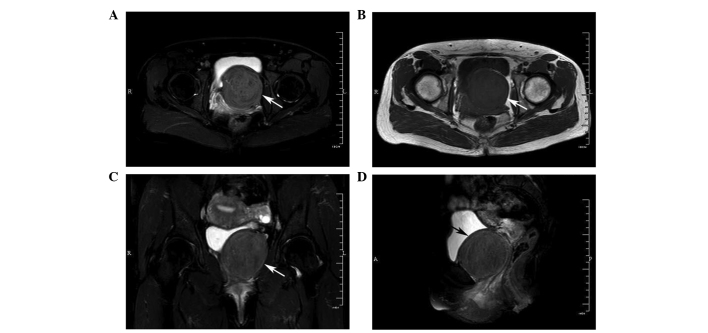
MRI showing the presence of the tumor on (A) T2-weighted and (B) T1-weighted images in the transverse plane. T2-weighted MRI showing (C) coronal and (D) sagittal planes. Arrows indicate the tumor. MRI, magnetic resonance imaging.

A 47-year-old Chinese female was admitted to the Department of Urology (The First Affiliated Hospital, College of Medicine, Zhejiang University, Hangzhou, Zheijiang, China), due to a solid mass detected during a gynecological examination. The patient was asymptomatic and had undergone two surgeries for urethral leiomyoma, which occurred six and nine years ago, respectively. Computed tomography demonstrated that the bladder and uterine were compressed by a 7.5×7.0-cm mass with well-defined outlines (Fig. 1). Magnetic resonance imaging was later performed to improve the definition of the structure and the association of the lesion with the urethra and vagina. The mass was isointense to muscle on T1-weighted images and slightly hyperintense on T2-weighted images, indicating a solid mass (Fig. 2).

**Figure 3:**
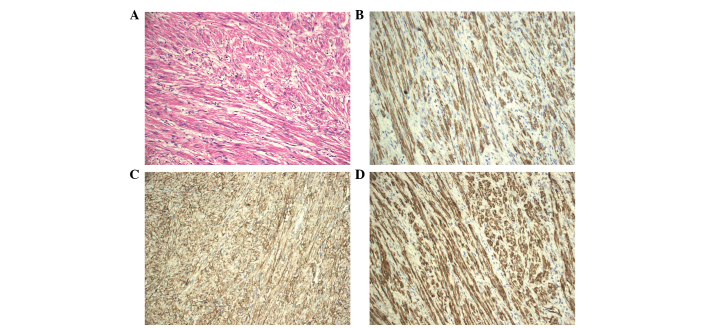
(A) Pathological results revealing the composition of the tumor, with spindle cells, a lack of nuclear atypia and mitotic figures (HE; magnification, ×200). Immunohistochemical analysis showing positive cytoplasmic staining for (B) desmin, (C) CD10 and (D) caldesmon in the tumor cells (magnification, ×200). HE, hematoxylin and eosin; CD10, cluster of differentiation 10.

The urethral tumor was completely excised and removed as close to the bladder neck as possible by transabdominal surgery. The detachment in the paraurethral region was meticulous to reduce injury to the urethra. The subsequent pathological diagnosis was of a leiomyoma. Immunohistological analysis demonstrated that the tumor cells were positive for desmin, cluster of differentiation 10 (CD10), smooth muscle actin and caldesmon, and negative for CD117 (Fig. 3). The patient was carefully followed up without any other treatment, and no sign of recurrence was observed in the first year post-surgery.

## Discussion

Leiomyomas are benign tumors of smooth muscle origin that occur throughout the genitourinary system, most commonly in the renal capsule (7). Urethral leiomyomas are rare benign tumors affecting females significantly more than males (1–8). In 1894, Buttner (10) described the first urethral leiomyoma. Thus far, only ~100 cases have been reported in the literature (1–8). The posterior wall of the urethra is the site of predilection, although any wall may be affected (11). Additionally, the distal urethra can be affected, but the proximal segment is the most common site (12).

Urethral leiomyomas are usually asymptomatic when they are small. As they grow in size, patients may complain of urinary tract infection, dyspareunia, urinary retention or irritative lower urinary tract symptoms (13). Physical examination may reveal a mass in the anterior vaginal wall or one that protrudes from the urethral meatus (12). Ultrasonography and magnetic resonance imaging have been shown to provide useful pre-operative information regarding the morphology and structure of the mass (14,15). However, a pathological examination is indispensable to exclude the possibility of a malignancy.

In the present study, the huge pelvic tumor should be differentiated from the urethral carcinoma or masses that have originated from other tissues, although the patient had a past history of urethral leiomyoma present six and nine years ago, respectively. In all previously published cases, the urethral leiomyomas have been treated surgically, with surgical excision as the first choice of treatment. The present patient was treated by transabdominal excision and the tumor was found to originate from the proximal segment of the urethra, which is close to the bladder neck.

All previously reported vesical and urethral leiomyomas have followed a benign biological course (7), with only a single reported recurrence treated by a repeat excision (9). The present study is the second reported case of urethral leiomyoma recurrence, and the patient has undergone three surgical procedures to date.
